# Effects of ovarian endometriotic fluid exposure on fertilization rate of mouse oocytes and subsequent embryo development

**DOI:** 10.1186/1477-7827-11-4

**Published:** 2013-01-18

**Authors:** Waraporn Piromlertamorn, Ubol Saeng-anan, Teraporn Vutyavanich

**Affiliations:** 1 Division of Reproductive Medicine, Department of Obstetrics and Gynecology, Faculty of Medicine, Chiang Mai University, Chiang Mai, 50200, Thailand

**Keywords:** Blastocyst, Endometriotic fluid, Fertilization, Mouse oocytes

## Abstract

**Background:**

Accidental exposure of oocyte/cumulus complex to endometriotic fluid is not uncommon during oocyte retrieval. Only two studies were available on this subject and they gave conflicting results. In this study, we used a mouse model to evaluate the effect of controlled exposure of oocytes to ovarian endometriotic fluid.

**Methods:**

Mouse oocytes/cumulus complexes (n = 862) were divided into 4 groups, and were exposed to endometriotic fluid (group 1), pooled sera from subjects without endometrioma (group 2), phosphate-buffered saline (group 3), and fertilization medium (controls). After five minutes, oocytes were washed and inseminated. Embryo development was observed daily. The quality of hatching blastocysts was assessed by counting the number of inner cell mass (ICM) and trophectoderm (TE) cells.

**Results:**

The fertilization, cleavage and blastocyst formation rates in the four groups were not statistically different. The proportions of hatching/hatched blastocysts from fertilized oocytes in groups 1 and 2 were significantly lower than those in group 3 and controls (*P* = 0.015). Hatching blastocysts from all groups showed no significant difference in the number of ICM and TE cells.

**Conclusions:**

Exposure of mouse oocytes/cumulus complexes to endometriotic fluid had subtle detrimental effects on subsequent blastocyst development. However, one should be cautious in projecting the results of this study to contaminated human oocytes in a clinical setting.

## Background

The impact of an accidental exposure of oocyte/cumulus complex to endometriotic content on the fertilization rate and subsequent embryo development remains unknown. Our PubMed search revealed only two studies [1,2] on this subject, with conflicting results. Suwajanakorn *et al.*[1] found a significant decrease in fertilization rates in 38 patients, who had some of their oocytes accidentally exposed to endometriotic fluid, compared to non-exposed and control oocytes (67%, 78% and 71% in contaminated, non-contaminated and control oocytes, respectively). On the other hand, Khamsi *et al.*[2] reported no detrimental effect on the fertilization rate (36/60 or 60% vs. 28/50 or 56% in contaminated and non-contaminated oocytes from 14 patients). However, both studies were small, invol-ving only 85 and 60 exposed oocytes and 301 and 50 non-exposed oocytes, respectively. Moreover, they were retrospective and observational in nature. Hence, the contradictory results could be due to many uncontrolled factors, such as the amount and duration of exposure to endometriotic fluid. Although a randomized trial of an adequate size would be the best way to answer this question, it is unethical in clinical practice.

In this study, we used a mouse model to evaluate the effects of controlled exposure of oocytes/cumulus complexes to ovarian endometriotic fluid. The outcomes included the fertilization rate of exposed oocytes, further cleavage of zygotes, blastocyst development and the quality of hatching blastocysts, as assessed by the number of inner cell mass and trophectoderm cells.

## Methods

### Animals

Outbred International Cancer Research (ICR) mice were purchased form the National Animal Institute, Mahidol University, Bangkok, Thailand. They were kept at our Animal Husbandry Unit in a well-ventilated room at 25 ± 2°C, under 60-70% humidity and controlled 12-hour light/12-hour dark cycles. Before the experiment, the mice were left undisturbed for 5 days to avoid the effect of stress from transportation. The Animal Ethics Committees of the Faculty of Medicine, Chiang Mai University approved the use of mice in our study (protocol no. 4/2551).

### Gonadotropin stimulation and oocyte collection

Five to seven-week old ICR female mice were super-ovulated by an intra-peritoneal (IP) injection of ten units of pregnant mare serum gonadotropin (Sigma, St. Louis, MO, USA), followed 48 hours later by an IP injection of ten units of human chorionic gonadotropin (Pregnyl, Organon, Oss, The Netherlands). Approximately 16 hours after the second injection, the mice were sacrificed by cervical vertebrae dislocation. The oviducts were removed and placed in warm culture medium (Fertilization medium, Cook, Brisbane, Australia), that was previously equilibrated overnight in a humidified atmosphere of 6% CO_2_ in air. The oocytes/cumulus complexes (COCs) were released from the bulging site of the oviduct by tearing it with a 30-gauge needle. They were washed through two drops of fertilization medium to remove debris and blood. Oocytes/cumulus complexes from each mouse were divided to four groups.

### Exposure of oocytes/cumulus complexes to endometriotic contents

In the first group, oocytes/cumulus complexes were exposed to 150 μL drops (8–10 oocytes/drop) of undiluted endometriotic fluid under paraffin oil (Medicult, Jyllinge, Denmark) at 37°C for five minutes under an atmosphere of 6% CO_2_ in air. Endometriotic fluid was obtained from five patients by aspiration at the time of a laparoscopic cystectomy. The fluids were pooled together and kept refrigerated at −70°C. The pooled endometriotic fluid was warmed to 37°C before the experiment. Oocytes/cumulus complexes in the second group were exposed to pooled sera that were collected from five subjects, who had normal level of CA-125 and had no evidence of endometrioma on transvaginal ultrasound examination. The pooled sera were kept at −70°C and warmed to 37°C before use. The exposure was done for five minutes in a similar condition. Oocytes/cumulus complexes in the third group were similarly exposed to phosphate-buffered saline (PBS; Gibco, New York, NY). In the last group, oocytes/cumulus complexes were transferred into 150 μL drops of fertilization medium (control) under oil. After five minutes, the oocytes/cumulus complexes were removed and washed five times in fertilization medium, before they were transferred into 100 μL drops of the same medium, over-layered with oil in 35 × 10 mm dishes (Nunc, Denmark). All procedures were performed in an IVF chamber (HD Scientific, NSW, Australia) under a humidified atmosphere of 6% CO_2_ in air at 37°C.

### Sperm retrieval and capacitation

Two proven fertile male mice of the same strain, aged 10–12 weeks, were sacrificed one hour before oocyte collection. The cauda epididymis and vas deferens were identified, and removed from both testes. They were washed in warmed equilibrated fertilization medium, and transferred to a 400 μL drop of the same medium in a 35 × 10 mm culture dish at 37°C. Spermatozoa were gently squeezed out of the epididymis and vas deferens, using a watchmaker’s forceps. The residual tissues were discarded. The spermatozoa obtained from the two mice were pooled, and capacitation was allowed to proceed for 60 to 90 minutes at 37°C in an atmosphere of 6% CO_2_ in air. Sperm concentration was assessed, using a Makler counting chamber (Sefi-Medical Instrument, Haifa, Israel).

### In vitro fertilization and embryo culture

The insemination and embryo cultures were performed in a CO_2_ incubator (Forma 310, Thermo Fisher Scientific, MA, USA) under an atmosphere of 6% CO_2_, in air at 37°C. The insemination was carried out in 100 μL drops of fertilization medium under paraffin oil. The capacitated sperm suspension was gently added to the drops containing oocytes/cumulus complexes to yield a final motile sperm concentration of 2 to 3 × 10^6^/mL. The oocytes, with few layers of attached cumulus cells, were removed from the insemination drops after four hours of incubation. They were washed two times in cleavage medium (Cook, Brisbane, Australia), and cultured in groups of eight to ten in 10 μL drops of equilibrated medium. Seven hours after insemination, fertilization was ascertained by the presence of two pro-nuclei and two polar bodies, under an inverted microscope at × 200 magnification (Eclipse TE2000-U with Hoffman modulation contrast; Nikon, Tokyo, Japan). After 48 hours, the embryos were transferred into blastocyst medium (Cook) and cultured under the same condition for another 48 hours. The embryos were assessed once daily for development. On the last day of culture, blastocysts were classified either as good quality if they reached the hatching/hatched stage of development, or poor quality if they were at earlier stages of development.

### Differential staining of ICM and TE

The hatching blastocysts from each group were differentially stained using the protocol described by Pampfer *et al*. [3]. In brief, the blastocysts were transferred into 0.5% protease (Sigma) and incubated at 37°C for five to ten minutes to remove the zona pellucida. The zona-free blastocysts were washed three to four times in calcium and magnesium free phosphate-buffered saline (Gibco) with 0.1% bovine serum albumin (BSA; Sigma A9418), before exposing them to rabbit anti-mouse antibody (Sigma M 5774; concentration 1:50) for 30 minutes at 37°C. After washing the blastocysts were transferred into guinea pig complement serum (Sigma S 1639), with propidium iodide (Sigma P 4170) and bisbenzimide (Sigma B 2261) at 37°C for 10–15 minutes. The blastocysts were washed and transferred onto glass slides, and allowed to air dry. The slides were mounted in glycerol and examined under a Nikon E600 epifluorescence microscope, equipped with LUCIA FISH program (Laboratory Imaging, Prague, Czech Republic). The number of inner cell mass (ICM) and trophectoderm (TE) cells were counted and recorded.

### Statistical analysis

Statistical analysis was performed using the STATA program, version 8.2 (StataCorp, College Station, Texas, USA). Chi-square tests were used to compare further cleavage rates, blastocyst and hatching blastocyst formation in the four groups. Mean numbers of ICM and TE cells were compared by one-way ANOVA. A two-tailed *P* < 0.05 was considered statistically significant.

## Results

### Fertilization rates

Eight hundred and sixty two mature mouse oocytes, from sixty-five mice, were used in this study. They were exposed to pooled endometriotic fluid (group 1; 231 oocytes), pooled sera from five subjects without endometrioma (group 2; 207 oocytes), PBS (group 3; 211 oocytes), or fertilization medium (controls; 213 oocytes). The fertilization rate was not significantly different between the four groups (Table 1).

**Table 1:** Effects of endometriotic fluid exposure on oocyte fertilization rate, subsequent cleavage of the zygotes, and blastocyst formation *(n = 862 oocytes)*

**Outcome**	**Endometriotic fluid (%)**	**Serum (%)**	**PBS (%)**	**Medium (%)**	***P***^***a***^
*Fertilization*	100/231	105/207	110/211	110/213	0.199
	(43.3%)	(50.7%)	(52.1%)	(51.6%)	
*Cleavage rate*^*b*^	82/100	89/105	97/110	96/110	0.581
	(82.0%)	(84.8%)	(88.2%)	(87.3%)	
*Blastocyst formation*^*b, c*^	49/100	53/105	67/110	64/110	0.229
	(58.2%)	(49.0%)	(50.5%)	(60.9%)	
*Hatching/hatched blastocyst*^*b*^	10/100	9/105	22/110	23/110	0.015
	(10.0%)	(8.6%)	(20.0%)	(20.9%)	

^a^Chi-Square.

^b^Calculated from fertilized oocytes (zygotes).

^c^Early, partial, full, expanding, hatching and hatched blastocysts are included in “Blastocyst formation”.

### Embryo development

Fertilized oocytes in the four groups showed no significant difference in further cleavage rate and blastocysts formation (Table 1). The rate of blastocyst formation calculated from the number of inseminated oocytes was also not statistically different (49/231 or 21.2%; 53/207 or 25.6%, 67/211 or 31.8%; and 64/213 or 30.0% in groups 1, 2, 3 and controls, respectively; Chisquare 7.55. P = 0.056). However, the proportion of hatching and hatched blastocysts from fertilized oocytes that were previously exposed to pooled serum or endometriotic fluid was significantly lower than those in the controls or PBS group (Table 1).

### Blastocyst cell numbers and ICM/TE ratio

There was no significant difference in the mean number of cells in the inner cell mass or trophectoderm of hatching blastocysts in the three experimental groups and controls (Table 2).

**Table 2:** Mean number of cells in the inner cell mass (ICM) and trophectoderm (TE), and total number of cells (ICM + TE) in hatching mouse blastocysts in four different groups

	**Control**	**PBS**	**serum**	**endometriotic fluid**	***P***^**a**^
	***(n = 15)***	***(n = 19)***	***(n = 9)***	***(n = 10)***	
ICM	25.4 ± 6.0	20.7 ± 6.5	23.3 ± 4.8	18.7 ± 7.7	0.0555
TE	38.7 ± 7.4	38.6 ± 8.1	44.6 ± 9.7	37.3 ± 10.8	0.2745
Total	64.1 ± 8.1	59.3 ± 9.9	67.9 ± 11.7	55.9 ± 14.1	0.0653

Values are mean ± SD; ^a^one-way ANOVA test.

## Discussion

Accidental exposure of oocyte/cumulus complex to endometriotic fluid is not uncommon during oocyte retrieval. Unfortunately, there is scanty and contradictory data on this subject to counsel patients or guide us in our clinical practice. To avoid the ethical and practical problem of dealing with human oocytes and embryos, mice were used as the animal model to explore the effects of endometriotic fluid exposure on oocyte fertilization and subsequent embryo development. The mouse embryo culture system has been used for many years as a tool to monitor culture conditions for human *in vitro* fertilization [4]. Moreover, it is useful as a bioassay for evaluating the potential toxicity of environmental contaminants on human fertilization and early embryo development [5,6]. An exposure time of five minutes was chosen in this study because most oocytes/cumulus complexes would have been removed from the contaminated follicular fluid during oocyte retrieval within this period. Our experimental model could be considered as an extreme condition, because in real-life situations oocyte/cumulus complexes are exposed to endometriotic fluid mixed with follicular fluid/flushing medium for a much shorter duration. As there was no significant decrease in fertilization rate of oocytes exposed to endometriotic fluid, the effect of exposure to serial dilution of the fluid or shorter exposure time was not studied further.

In this study, oocytes/cumulus complexes were exposed to endometriotic fluid to assess its effect on fertilization and subsequent embryo development. In the control group, they were exposed to fertilization medium before insemination in the same medium. We included two more comparison groups as exposure of oocytes/cumulus complexes to another fluid/medium with different basic compositions, pH and osmolality from fertilization medium (pH 7.5-7.8, osmolality 285–295 mOsm/kg) could induce stress to the oocytes, and had an effect by itself on subsequent embryo development. In one group, oocytes/cumulus complexes were exposed to PBS, which had a pH of 7.4 ± 0.1 and an osmolality of 282–288 mOsm/kg. In another comparison group, we exposed them to pooled sera from normal subjects without endometrioma. The rationale was that endometriotic fluid was a collection of old blood. Its composition should, therefore, be similar to serum with less water and the presence of degenerated red blood cells, inflammatory cells, and other possible toxic products to oocytes/early embryos. We treated the pooled sera the same way as endometriotic fluid, in terms of storage, thawing, and exposure to oocyte/cumulus complexes. If endometriotic fluid did contain some toxic factors, we expected that oocyte/cumulus complexes exposure would show significant decrease in fertilization and/or cleavage and/or blastocyst formation rate compared to those exposed to pooled sera from subjects without endometrioma. The fact that there was no difference in the developmental competence of oocytes that were exposed to endometrioma (group 1) or pooled sera of subjects without endometrioma (group 2), suggested that there was no direct influences of the endometriotic content *per se*. The significant decrease in the proportion of hatching/hatched blastocysts in these two groups, compared to those exposed to fertilization medium (control) and PBS (group 3), could be due to nonspecific effects common to both the endometriotic fluid and pooled sera, such as suboptimal pH and osmolality of the fluid [7,8] as both of them were not equilibrated with 6% CO_2_ or adjusted for osmolality before use.

The fertilization rates of 52.1% and 51.6% in oocytes/cumulus complexes that were previously exposed to PBS and fertilization medium (control) were compatible with a previous report by Vergara et al. [9], for this strain of mouse (52%), using a similar superovulation protocol and culture conditions. Our blastocyst formation rate of 50-60% and the mean (±SD) number of cells in our blastocysts (55–64 cells) was also in line with other previous reports for ICR mouse blastocysts that were produced *in vitro* (blastulation rate of 27%-54% and blastocyst cell count of 22–84) [9-12].

Our study (n = 200 oocytes/group) was designed to detect a 25% decrease in fertilization rate of oocytes after exposure to endometriotic fluid, with a power of 80% and a type-I error of 5% (two-tailed). A smaller decrease in fertilization rate of 10% or less, as suggested by Suwajanakorn *et al.*[1], could have gone unnoticed. In agreement with Suwajanakorn *et al.*[1] and Khamsi *et al.*[2], we did not observe a detrimental effect of endometriotic fluid on cleavage rate of the zygotes or the formation of good-quality embryos during our daily observation of embryo development. We also found no significant difference in the blastulation rate between the exposed and non-exposed oocytes, which was not reported in the two previous studies. However, the proportion of hatching and hatched blastocysts was significantly lower in the fertilized oocytes that were previously exposed to endometriotic fluid. It was reassuring that hatching blastocysts from all experimental groups and controls had no significant difference in the number of inner cell mass or trophectoderm or total cells. However, the implantation and pregnancy potential of these blastocysts could not be determined, as we did not transfer them into the uterus.

## Conclusions

Our data suggested that the exposure of oocytes/cumulus complexes to endometriotic fluid for five minutes had no detrimental effect on the fertilization rate and further cleavage of the zygotes. Hatching and hatched blastocyst formation was reduced, but the number of cells in the hatching blastocyst was not impaired. However, one should be very cautious in projecting the results of this study to contaminated human oocytes in a clinical setting.

## Abbreviations

ICM: Inner cell mass; TE: Trophectoderm; μL: Microliter.

## Competing interests

The authors declare that they have no competing interests.

## Authors’ contributions

WP: data collection and performing the statistical evaluation, and critical revision of the manuscript. US: data collection, and approval of the final version of the manuscript. TV: conception and design, interpretation and analysing the data, and writing the manuscript. All authors read and approved the final manuscript.
